# Age at detection and age at presentation of childhood cataract at a tertiary facility in Ibadan, Southwest Nigeria

**DOI:** 10.1186/s12886-020-1323-7

**Published:** 2020-01-30

**Authors:** Bolutife Ayokunnu Olusanya, Mary Ogbenyi Ugalahi, Adegbola Oluwagbemiga Adeyemo, Aderonke Mojisola Baiyeroju

**Affiliations:** 1 Department of Ophthalmology, College of Medicine, University of Ibadan, Ibadan, Nigeria; 2 Department of Ophthalmology, University College Hospital, Ibadan, Nigeria

**Keywords:** Childhood, Cataract surgery, Access, Nigeria, Sub-Saharan Africa

## Abstract

**Background:**

To describe factors which influence the age at detection and age at presentation of patients with childhood cataract at a tertiary eye care facility in Southwest Nigeria.

**Methods:**

A retrospective review of children who presented with cataract between 2011 and 2015. Case notes were reviewed and data on age at detection and presentation as well as other clinical information was collected and analyzed using Stata 12 statistical software.

**Results:**

A total of 164 cases were reviewed, 52.4% of them were boys. Median age at presentation was 48 months while the median age at detection was 13.5 months. Seventy-four (45.1%) children had congenital cataract, 31.1% had developmental cataract, and 21.3% had traumatic cataract. The child’s mother detected the cataract in 116 (70.7%) of the patients. Median age at presentation for patients with congenital cataract was 18 months and 84 months for developmental cataract. The median age at presentation for congenital cataracts that were noticed by the mother was 17 months compared with 72 months for those noticed by other caregivers (*p* = 0.0085). The median age at presentation for developmental cataracts that were noticed by the mother was 72 months compared with 114 months for those noticed by other caregivers (*p* = 0.0065). Gender of the child did not significantly influence the age at detection or presentation. The source of referral and the location of domicile did not significantly affect the time interval between detection of the cataract and presentation to hospital.

**Conclusion:**

The average age of children presenting with cataracts in our setting is older than in high income countries. Detection of the cataract by the mother increases the likelihood of early presentation; thus, focused maternal education may promote earlier detection and presentation.

## Background

Childhood cataract is a major cause of childhood blindness and impaired vision worldwide. In low income countries there is a high burden of childhood cataract with a child becoming bilaterally blind every minute [1].

Blindness from childhood cataract is a huge challenge in terms of economic loss; social burden and morbidity [1], with an estimate of about 200,000 children blind from cataract worldwide and about 20,000–40,000 children born each year with congenital cataract [2]. The incidence however varies across regions worldwide. A study conducted in West Africa in schools for the blind had 15.5% of blindness due to lens related abnormalities [3], compared to 35% in Malawi (Southern Africa), 33% in Ethiopia (Eastern Africa), 9.2% in Chile (South America) and 7.4% in South India (Asia) [4, 5].

Cataract is the most important cause of treatable childhood blindness [2]. Outcome of treatment is influenced by the time of presentation of the patient, time of cataract surgery and prompt optical rehabilitation; with a higher risk of stimulus deprivation amblyopia when treatment is delayed [1]. Other factors affecting outcome include the quality of the surgery as well as prompt and appropriate management of complications occasioned by long-term follow up [6].

Previous reports have demonstrated that delayed presentation for treatment among children with childhood cataract is a common phenomenon in low income countries [7, 8]. Various factors including socio-demographic, cultural and health system factors have been associated with delayed presentation. There is a need to investigate the reasons for such delay in our environment [9]. The aim of this study was to determine the factors which influenced the age at detection and the age at presentation of patients with childhood cataract at a child eye health tertiary facility in Ibadan, Southwest Nigeria. We also investigated factors which may influence the timing of presentation, specifically the length of time between the detection of the cataract and presentation to hospital.

## Methods

This was a retrospective review of all patients with childhood cataract seen in our hospital over a five-year period (2011 to 2015). Children aged 0 to 16 years who presented with cataracts during the study period were identified from the diagnosis register of the eye clinic. Their case records were retrieved and relevant information including biodata, age at detection of the main presenting complaint (e.g. white speck in the eye(s) or poor vision), age at presentation to our hospital and relationship of the person who first noticed the cataract (i.e. the main presenting complaint) were extracted. Other information noted were the source of referral, place of domicile, and type of cataract. Children with missing or incomplete records were excluded from the study. The study adhered to the tenets of the Declaration of Helsinki.

The information collected was stored in a secure manner with no patient identifier to ensure confidentiality and data was analyzed with Stata 12 statistical software. Summary indices were generated for univariate analysis. These indices were in the form of frequency distributions for categorical variables and measures of central tendency (mean, median) as well as measures of dispersion (standard deviation, interquartile range) for the quantitative variables. Bivariate analysis was conducted with the use of cross tabulations and chi-square test to evaluate associations between categorical variables. The Mann Whitney test was used to evaluate associations between quantitative and categorical variables. Any *p* value less than 0.05 was considered to be statistically significant.

For the purpose of this study, early presentation was defined as presentation to hospital within 3 months of detection of the main presenting symptom for congenital cataracts and 6 months for developmental and other types of cataracts.

## Results

A total of 193 children were identified to have presented with cataracts during the study period. Of these, the records of 29 children were either missing or incomplete, thus 164 patients were included in this study. All the patients were domiciled in Southwestern Nigeria with 111 (67.7%) residing in Ibadan, Oyo state where the hospital is located. Males accounted for 90 (54.9%) of the children. The median age of the children at presentation to our facility was 48 months with a range of 2–180 months while the median age at detection was 13.5 months with a range of 0–156 months.

The cataract was detected by the mother in 116 (70.7%) children, by the father in 23 (14%) children and the school teacher in seven (4.3%) children. Seven (4.3%) children were reported to have detected the cataract by themselves. For the remaining 11 (6.7%) children, the cataract was detected by other people, including primary health care worker (1), grandmother (5), aunt (4), and care giver at orphanage (1).

Seventy-four (45.1%) of the patients had congenital cataracts, 51(31.1%) had developmental cataract, and 35 (21.3%) had traumatic cataract. Four patients (2.4%) had other types of cataract including Uveitic cataract (3) and Marfan syndrome (1). Among the children with congenital cataracts, 44 (59.5%) were detected at birth. The median age at detection for congenital cataract was 0 months (i.e. detected at birth) with an interquartile range (IQR) of 5 months, while the median age at presentation for congenital cataract was 18 months (IQR = 32 months). With regards to developmental cataract, median age at detection was 54 months (IQR = 74 months) while the median age at presentation was 84 months (IQR = 72 months).

The source of referral was from other public (government-run) hospitals for 91 (55.5%) children while 14 (8.6%) patients presented primarily to our clinic without any referral. Thirty-one (18.9%) children were referred by private or mission hospitals and 28 (17.1%) of the children were referred by the outreach unit of our clinic during screening camps in the community.

We evaluated the effect of gender on the age at detection and age at presentation (Table 1). The median age at which congenital cataract was detected was the same (i.e. at birth) for both males and females (*p* = 0.3308), however the boys who had congenital cataract were brought to hospital at a younger age compared to girls (*p* = 0.2524) (Table 1). Developmental cataracts were detected at a younger median age in girls (*p* = 0. 4692), but the median age at presentation was younger in boys compared to girls (*p* = 0. 7038) (Table 1). For traumatic cataracts, the median ages at detection and at presentation were the same for both males and females (Table 1).

**Table 1:** Effect of gender on age at detection and age at presentation of patients

Cataract type (Number)	Male	Female	*P* value*
	Median age (months)	Median age (months)	
Age detected			
Congenital (74)	At birth (IQR^a^ = 3)	At birth (IQR = 5)	0.3308
Traumatic (35)	96 (IQR = 60)	96 (IQR = 72)	0.9713
Developmental (51)	60 (IQR = 72)	48 (IQR = 75)	0.4692
Age presented			
Congenital (74)	14 (IQR = 30)	19.5 (IQR = 30)	0.2524
Traumatic (35)	96 (IQR = 72)	96 (IQR = 72)	0.6260
Developmental (51)	84 (IQR = 72)	90 (IQR = 60)	0.7038

^a^*IQR* Interquartile range

*Mann Whitney test

Furthermore, we investigated whether the age at detection or age at presentation was associated with the person who detected the cataract (Table 2). For congenital cataracts that were detected by mothers, the median age at detection was “at birth”; this was the same as the median age for congenital cataracts detected by other caregivers (*p* = 0.2806) [Table 2]. For developmental cataracts, the median age at detection by mothers was 48 months, while the median age at detection by other caregivers was 72 months (*p* = 0.2408) (Table 2). The median age at presentation of children with congenital cataracts detected by mothers was younger than for congenital cataracts detected by other caregivers (*p* = 0.0085) (Table 2). Similarly, children with developmental cataracts detected by mothers had a younger median age at presentation compared to those whose cataracts were noticed by other caregivers (*p* = 0.0065) (Table 2).

**Table 2:** Effect of person who detected the cataract on age at detection and age at presentation of patients

Cataract type (Number)	Mother	Others	*P* value*
	Median age (months)	Median age (months)	
Age detected			
Congenital (74)	At birth (IQR^a^ = 6)	At birth (IQR = 1)	0.2806
Traumatic (35)	84 (IQR = 51)	120 (IQR = 60)	0.0470
Developmental (51)	48 (IQR = 81)	72 (IQR = 54)	0.2408
Age presented			
Congenital (74)	17 (IQR = 28)	72 (IQR = 97)	0.0085
Traumatic (35)	96 (IQR = 54)	120 IQR = (48)	0.0236
Developmental (51)	72 (IQR = 60)	108 (IQR = 114)	0.0065

^a^*IQR* Interquartile range

*Mann Whitney test

Only 55 (33.5%) of the children presented early i.e. within 3 months of detection (for congenital cataract) or within 6 months (for other types of cataract). Early presentation was noted in 21 (28.4%) of the children with congenital cataract, and 10 (19.6%) of those with developmental cataracts, while 24 (68.6%) of those with traumatic cataracts presented early (*p* < 0.001). Further analysis into factors associated with early versus late presentation revealed that among children with congenital cataracts, those whose mother detected the cataract were more likely to present early when compared to the children whose cataract was detected by other caregivers (Odds ratio [O.R.] = 5.24; 95% Confidence Interval [C.I.] = 0.63–43.43; *p* = 0.09) (Table 3). There was a similar finding among the children with developmental cataracts (O.R. = 2.56; 95% C.I. = 0.48–13.62; *p* = 0.26) (Table 3).

**Table 3:** Effect of relationship of person who detected cataract on early presentation

		Presentation			
Type of cataract (Number)	Person who noticed	Early Number (%)	Late Number (%)	Odds ratio (95% C.I.)^a^	*P* value*
Congenital (74)	Mother	20 (32.3%)	42 (67.7%)	5.24 (0.63–43.43)	0.09
	Others	1 (8.3%)	11 (91.7%)		
Developmental (51)	Mother	8 (24.2%)	25 (75.8%)	2.56 (0.48–13.62)	0.26
	Others	2 (11.1%)	16 (88.9%)		

^a^95% C.I. – 95% Confidence Interval

* Chi-square test

With regards to gender, males with congenital cataract were more likely to present early compared to females (O.R. = 2.12; 95% C.I. = 0.75–5.97; *p* = 0.15) (Table 4). Regarding the association between state of domicile and early presentation for congenital cataracts, we found that children living in Oyo state, where our hospital is located, were less likely to present early (O.R. = 0.63; 95% C.I. = 0.22–1.78; *p* = 0. 38) (Table 5). Similarly, we observed that children with congenital cataracts who were referred by the outreach unit were less likely to present early (*p* = 0.98) (Table 6).

**Table 4:** Effect of gender on early presentation

		Presentation			
Type of cataract (Number)	Gender	Early Number (%)	Late Number (%)	Odds ratio (95% C.I.)^a^	*P* value*
Congenital (74)	Male	13 (36.1%)	23 (63.9%)	2.12 (0.75–5.97)	0.15
	Female	8 (21.1%)	30 (78.9%)		
Developmental (51)	Male	6 (26.1%)	17 (73.9%)	2.12 (0.52–8.67)	0.29
	Female	4 (14.3%)	24 (85.7%)		

^a^95% C.I. – 95% Confidence Interval

* Chi-square test

**Table 5:** Effect of state of domicile on early presentation

		Presentation			
Type of cataract (Number)	State of domicile	Early Number (%)	Late Number (%)	Odds ratio (95% C.I.)^a^	*P* value*
Congenital (74)	Oyo state	12 (25.0%)	36 (75.0%)	0.63 (0.22–1.78)	0.38
	Other states	9 (34.5%)	17 (65.4%)		
Developmental (51)	Oyo state	6 (18.8%)	26 (81.3%)	0.87 (0.21–3.57)	0.84
	Other states	4 (21.1%)	15 (78.9%)		

^a^95% C.I. – 95% Confidence Interval

* Chi-square test

**Table 6:** Effect of source of referral on early presentation

		Presentation		
Type of cataract (Number)	Source of referral	Early Number (%)	Late Number (%)	*P* value*
Congenital (74)	Public hospital	12 (28.6%)	30 (71.4%)	0.98
	Private/Mission hospital	4 (30.8%)	9 (69.2%)	
	Eye Outreach unit	2 (22.2%)	7 (77.8%)	
	Self-referral	3 (30%)	7 (70%)	
Developmental (51)	Public hospital	6 (20%)	24 (80%)	0.36
	Private/Mission hospital	3 (33.3%)	6 (66.7%)	
	Eye Outreach unit	1 (8.3%)	11 (91.7%)	
	Self-referral	Nil	Nil	

* Chi-square test

## Discussion

More than half of our patients were boys which is comparable to results from studies in Kenya [10], Madagascar [11], Tanzania [7] and China [12] in which two thirds of the cases were boys. The average age at presentation of children with cataracts in our setting is similar to age at presentation of children with cataracts from other low and middle income countries like ours [10, 11]. The overall median age of 4 years at presentation reported from our study is, however, older than that reported from United Kingdom, a high income country with screening services for childhood cataract where majority of the patients were diagnosed before 1 year of age [13]. Though the median age of presentation for congenital cataract was 18 months, we observe that children in our environment presented later than reported from a high income country [13].

We observed that although the age at detection was the same for both boys and girls with congenital cataracts, there was a tendency for boys to present to hospital at an earlier age than girls. This may suggest that girls with cataract in our environment may not access cataract services promptly. It has been reported that access to surgery for childhood cataract is poorer among females compared to males worldwide and this it is particularly worse in Sub-Saharan Africa, East Asia, South Asia and the Pacific [6, 14, 15]. A study from Iran observed more males presenting earlier for second eye surgeries as compared to females [16]. Thus, even after presentation, uptake may still be affected by the gender of the child.

In majority of the cases, the cataract was noticed by the mother. This is not unexpected in view of mother- child relationship/bonding, particularly in early childhood. The age at detection of congenital cataract was the same regardless of the person who noticed it. However, children whose mothers had detected the cataract were more likely to present within 3 months of detection and also presented at a younger age. This was the case particularly with regards to congenital cataracts which have a higher risk of poor visual outcome when surgical intervention is delayed.

This finding suggests that education and empowerment of mothers may have a positive impact on early detection and presentation for childhood cataract in our environment. Specifically, focused maternal education about ways of detecting childhood cataract and the need for early presentation and treatment may promote earlier detection and presentation. This should have a long term effect of reducing blindness from childhood cataract in resource poor countries where screening programmes are few or non-existent.

Distance from the hospital has been identified as a significant factor for late presentation in children with cataracts [7]. On the contrary, in our study we found that a larger proportion of children living within the state where the hospital is located presented late compared with those residing in other states. Although this finding was not statistically significant, it may suggest that children living closer to treatment centres may present later for treatment. This highlights the fact that other social issues may be responsible for late presentation and further research, using qualitative methods, is necessary to find out the hidden reasons why children are brought late for cataract surgery in Nigeria.

It is imperative to note that almost one-fifth of the referrals were from the outreach unit of the hospital. This suggests that the current healthcare structure is not all-encompassing and a significant proportion of the population are still unreached, that is, the segment of the population that may not seek orthodox medical care until healthcare personnel go to their communities. Efforts at establishing sustainable case detection mechanisms in the communities will ensure early detection and referral to treatment centres. Moreover, interventions that involve the integration of screening into primary health care activities such as immunisation are likely to be quite useful in ensuring early detection [17]. Furthermore, proper counselling of parents/ caregivers of children referred from primary care level and outreach programmes is necessary to encourage early presentation for treatment at the base hospital.

The limitations of this study include its retrospective nature and the fact that we did not explore socioeconomic status and education of parents as factors associated with age at detection or presentation. Another limitation is the fact we were unable to investigate the effect of cataract morphology on detection and presentation. A prospective study using a mixed methods design that involves in-depth interviews of mother and other caregivers such as grandmothers could shed more light on the factors that influence the detection and presentation of childhood cataracts in our setting.

## Conclusion

The average age of children presenting with cataracts in our setting is older than in high income countries and majority of them present late for treatment. Such late presentation among Nigerian children is a cause for grave concern because of its negative impact on treatment outcome. Education of mothers and other caregivers about childhood cataract and the need for early presentation and treatment may encourage earlier presentation among our population.

## Data Availability

The dataset analysed are available with the corresponding author and may available on request after appropriate permission is given by the hospital ethical committee for sharing.

## Abbreviations

C.I.: Confidence interval
IQR: Interquartile range
O.R.: Odds ratio
